# Cost and cost-effectiveness of the ‘Stand and Move at Work’ multicomponent intervention to reduce workplace sedentary time and cardiometabolic risk

**DOI:** 10.5271/sjweh.4022

**Published:** 2022-06-30

**Authors:** Tzeyu L Michaud, Wen You, Paul A Estabrooks, Krista Leonard, Sarah A Rydell, Sarah L Mullane, Mark A Pereira, Matthew P Buman

**Affiliations:** 1 Center for Reducing Health Disparities, College of Public Health, University of Nebraska Medical Center, Omaha, NE, USA; 2 Department of Health Promotion, College of Public Health, University of Nebraska Medical Center, Omaha, NE, USA; 3 Department of Public Health Sciences, University of Virginia, Charlottesville, VA, USA; 4 Department of Health and Kinesiology, University of Utah, Salt Lake City, UT, USA; 5 College of Health Solutions, Arizona State University, Phoenix, USA; 6 Division of Epidemiology and Community Health, University of Minnesota, Minneapolis, USA; 7 Johnson & Johnson Health and Wellness Solutions, Inc., High Wycombe, England, United Kingdom

**Keywords:** absenteeism, cost-benefit, presenteeism, sit-stand workstation, social-ecological framework, workplace health promotion

## Abstract

**Objective:**

Few studies have reported the cost and cost-effectiveness of workplace interventions to reduce sedentary time. The purpose of this study was to complete an economic evaluation of a multilevel intervention to reduce sitting time and increase light-intensity physical activity (LPA) among employees.

**Methods:**

We conducted a retrospective within-trial cost and cost-effectiveness analysis (CEA) to compare a 12-month multilevel intervention with (STAND+) and without (MOVE+) a sit-stand workstation, across 24 worksites (N=630 employee participants) enrolled in a cluster randomized clinical trial. We estimated the intervention costs using activity-based costing strategy. The intervention costs were further expressed as per person and per worksite. CEA was conducted using an incremental cost-effectiveness ratio (ICER) metric, expressed as costs for additional unit of sitting time (minute/day), LPA (minutes/day), cardiometabolic risk score, and quality-adjusted life years (QALY) increased/decreased at 12 months. We assessed the cost analysis and CEA from the organizational (ie, employer) perspective with a one-year time horizon.

**Results:**

Total intervention costs were $134 and $72 per person, and $3939 and $1650 per worksite for the STAND+ (N worksites = 12; N employees = 354) and MOVE+ (N worksites = 12; N employees = 276) interventions, respectively. The ICER was $1 (95% CI $0.8–1.4) for each additional minute reduction of workplace sitting time (standardized to 8-hour workday); and $4656 per QALY gained at 12 months. There was a modest and non-significant change of loss of work productivity improvement (-0.03 hours, 95% CI -4.16–4.09 hours), which was associated with a $0.34 return for every $1 invested.

**Conclusions:**

The multi-level intervention with sit-stand workstations has the potential to be widely implemented to reduce workplace sitting time. Future research into work productivity outcomes in terms of cost-benefits for employers is warranted.

Sedentary behaviors (SB), ie, sitting/lying at low energy expenditure while awake, are established modifiable risk factors of all-cause mortality and cardiometabolic outcomes [eg, obesity, diabetes, high blood pressure, high-density lipoprotein (HDL) cholesterol] (1–3), and subsequently cause substantial economic burden to society. For example, using a top-down approach and a population attributable fraction formula, Ding et al (4) estimated that the global cost of physical inactivity is $67.5 billion (international dollars; across coronary heart disease, stroke, type 2 diabetes, breast cancer, and colon cancer), a result of associated increased healthcare utilizations and loss of productivity due to mortality. Using a similar approach, Heron et al (5) estimated the cost of physical inactivity to be £0.8 billion (including type 2 diabetes, cardiovascular disease, colon cancer, endometrial cancer and lung cancer) in the UK. Thus, SB have emerged as an important public health concern. SB in office workers are of particular interest given desk-based workers spend approximately 77% of their workday as sedentary (6, 7), potentially due to advances in technology and computer-based tasks (8).

In response, an increasing number of multi-level/multi-component workplace SB interventions have been developed targeting individual, social, and environmental factors of SB. These types of interventions are distinct from physical activity interventions and target reductions in sitting time rather than increases in physical activity. Several reviews have suggested that workplace SB interventions, particularly those that implement the use of sit-stand workstations, can effectively reduce workplace SB by >60 minutes per 8-hour workday in 3–9 months (9–11). Workplace SB reduction interventions may also be beneficial from an organizational standpoint. Specifically, in addition to SB, researchers have found that reduced SB following a standing intervention does not appear to come at the expense of productivity or performance outcomes (eg, executive function or memory) (7, 12, 13).

Cost-effectiveness analyses (CEA) of multilevel workplace SB reduction interventions may be critical for workplace adoption decision-making, program implementation, and sustainability. Notably, Gao et al (14) applied a simulation modeling approach to examine the cost and cost-effectiveness of the “Stand Up Victoria” intervention (15, 16), a multi-component intervention that incorporated environmental, organizational, and individual-level strategies to reduce workplace sitting time. They found that “Stand Up Victoria” was cost-effective compared to usual practice. Similarly, Munir et al (17) examined the cost-benefit of Stand More AT Work (SMArT Work), a multi-component workplace SB reduction intervention that incorporated a sit-stand workstation and supporting behavior change strategies, and found cost-savings due to increases in productivity. In contrast, after examining the cost-effectiveness and return-on-investment of a sitting reduction workplace intervention (Dynamic Work), Ben et al (18) showed that the intervention may be cost-beneficial for the employers with a positive return on investment of $4.2 but the finding was not statistically significant. Finally, a recent review (19) including 18 economic evaluations of workplace interventions to improve SB and/or improve physical activity reported that although the work productivity-associated costs were the main cost-driver, the economic evidence is still unknown given that included interventions were heterogeneous, overall effects were small, and the interventions’ impact on costs was unclear. Further, the authors concluded that there is a need for more evidence and particularly long-term cost-effectiveness evidence.

Recently, the Stand & Move at Work (SMW) trial examined SB and cardiometabolic risk following a multi-level behavioral intervention that incorporated workplace policy, environmental, social, and individual-level strategies with (STAND+) and without (MOVE+) a sit-stand workstation (20). The SMW intervention resulted in a difference of approximately 60 minutes per 8h workday in sitting time at 12-months, favoring the STAND+ intervention. Although not significant, there were also differences in light-intensity physical activity (LPA) and clustered metabolic risk scores, favoring the STAND+ intervention (21). The purpose of this paper is to report on the cost and cost-effectiveness of implementing the 12-month SMW intervention from an organizational perspective.

## Methods

### Study design

Using retrospective data collected during the SMW trial (January 2016 to December 2017), we estimated the costs of implementing the multi-level SB intervention. We conducted a CEA following the recommendations of the Trial-Based Economic Evaluations in Occupational Health (22), and Consolidated Health Economic Evaluation Reporting Standards (23).

### Setting

The design and outcomes related to the overall study have been described in detail elsewhere (20, 21, 24, 25). In brief, the SMW cluster randomized trial was conducted in 24 worksites across academic, industry/healthcare, and government sectors in the Minneapolis/Saint Paul (Minnesota) and Phoenix (Arizona) regions (4 worksites in each region/sector spectrum), with 630 employees enrolled between January 2016 and November 2016. Eligibility criteria of worksites included: (i) small-to-moderate workgroup size (ie, 20–60 employees); (ii) >80% of employees working full time; (iii) predominantly seated desk-based office work; (iv) not currently undergoing a worksite wellness program to reduce sitting or increase LPA; (v) <10% of employees using a sit-stand workstation; (vi) willing to have sit-stand workstations installed; and (vii) leadership willing to be randomized to either study arm (20). Eligibility criteria for potential employee participants were: (i) ≥18 years; (ii) generally good health and able to safely reduce sitting and increase LPA; (iii) working full-time on-site; (iv) not currently pregnant; (v) predominant worksite occupation requiring seated office work; (vi) not currently using a sit-stand workstation; (vii) willing to have a sit-stand workstation installed at their desk; and (viii) willing to be randomized to either study arm (21). Eligible worksites were randomized to either a multi-level behavioral intervention (MOVE+, 12 sites with 354 participants) or a multi-level behavioral intervention along with newly installed sit-stand workstations (STAND+; 12 sites with 276 participants). The primary outcomes were time spent sitting and in LPA at work at 12-months. The majority of study participants were non-Hispanic white (72%), had college/some college education (63%), and had a professional job type (56%). Participants were distributed equally across work sectors at baseline. All worksites were retained with 487 participants (N=263 for STAND+ and N=224 for MOVE+) completing the 12-month intervention and assessment (21). Ethical approval for the SMW trial was obtained from the Arizona State University and the University of Minnesota Institutional Review Boards and was registered at clinicaltrials.gov (NCT02566317).

### Stand & Move at Work program

SMW was a workplace intervention designed to accompany sit-stand workstations to reduce sedentary time and increase LPA. It was grounded in a social-ecological framework incorporating individual (education, behavioral cues, goal setting), social (contests, role modeling), environmental (signage, centrally located waste bins), and organizational (managerial support, worksite sponsored messaging) strategies. The implementation of SMW was dependent on the identification and engagement of worksite administrators and managers to enact policy-level changes, implement environmental changes (eg, workplace signages or sit-stand workstations), and model and promote behavior change (20). Thus, worksites were required to identify a worksite leader and advocate(s). Advocates played an active role with study participants and were the chief implementers of the intervention. Leaders were administrative employees who provided support for the intervention strategies to be implemented. The primary difference between the STAND+ and MOVE+ intervention arms was the incorporation of sit-stand workstations within STAND+ worksites only.

### Perspective and time horizon

We assessed the cost analysis and CEA of the SMW program from the organizational (ie, employer) perspective given that the employers make the decision of whether or not to adopt or sustain the program after the grant funding ceases. We conducted the analyses over a one-year time horizon and thus the costs and effects were not discounted.

### Cost measures (SMW intervention delivery costs)

Following the cost assessment procedure of behavioral interventions proposed by Ritzwoller et al (26), the cost analysis of SMW consisted of five elements: (i) perspective of the analysis, (ii) identifying costs components, (iii) capturing relevant costs, (iv) data analysis, and (v) sensitivity analysis. We calculated costs by applying standard unit costs [ie, national average hourly wage rate (27)] to the quantity of resources used. All costs were expressed as 2020 US dollars ($), using the Consumer Price Index (28).

We divided intervention delivery costs into labor costs and non-labor costs. We estimated labor costs based on time spent on each intervention activity. Specifically, we utilized a micro-costing approach with an activity-based costing strategy (29) for cost estimates of the SMW intervention where the majority of multi-level behavioral change strategies to enhance sources of self-efficacy, improve outcome expectations, and leverage person-environment-behavior interactions are activity-based. We identified associated resources used to deliver the intervention via detailed intervention elements, categorized into policy-, organizational-, environment-, individual- and social-levels, defined in the study protocol (20). Specifically, we interviewed two advocates across different worksites, project coordinators, and staff to retrospectively provide estimates on the amount of time they invested on each activity for the behavioral change strategies. Moreover, we supplemented the cost data collection with meeting attendance logs and notes to identify the associated personnel for the activity. Table 1 outlines the associated activities for each behavioral change strategy, involved personnel, quantity of resources, and the estimated times for a specific activity by these levels. We calculated the costs associated with each activity by multiplying the total time (in hours) spent on that activity by the unit cost (eg, national average hourly wage) of the personnel conducting the activity. All research-related activities (eg, time on protocol development and intervention assessment) were excluded. We assumed one leader and one advocate per worksite (because intervention activities did not increase by the number of advocates involved and the majority of worksites only included a single advocate), where the unit cost for the leaders and advocates has a value of $27.07 per hour, the national average hourly wage in 2020 (27). The local hourly rate for master-level project staff to facilitate with the program implementation (eg, the workstation installation and training) was set at $16.35 per hour.

**Table 1:** Cost estimates of the Stand & Move at Work intervention delivery by study arms.

Intervention strategy	Associated activity	Personnel involved ^a^	Quantity	STAND+ (N=354)		MOVE+ (N=276)	
	
				Total hour	Total cost ($)	Total hour	Total cost ($)
Policy level	Determine appropriate toolkit items	Advocate	1.5 hr × 4 times × 12 sites	72	1949	72	1949
	Encourage participation in worksite initiatives	Advocate	30 min/month × 12 months × 12 sites	72	1949	72	1949
Organizational level	Quarterly support email sent by leaders	Leader	5 min × 4 times × 12 sites	4	108	4	108
	Quarterly meeting- review past progress and plan for the coming quarter	Advocate & project coordinator	45 min × 4 times × 12 sites	72	1949	72	1949
	Quarterly audit	Advocate & project coordinator	30 min × 4 times × 12 sites	24	1299	24	1299
	Monthly conference call with the PI	Advocate & PI	1 hr/month × 10 months × 6 sites	120	3248	120	3248
Environment level	Conduct workspace inventory for worksites	Project staff & worksite facility reps (N=2)	1 hr × 4 times × 12 sites	144	3384	144	3384
	Workstation installation ^b^	Project staff	30 min/participant	178	2894		
	Coaching session for workstation ^b^	Project staff	15 min/participant	89	1447		
	Create walking route	Advocate	10 min × 12 sites	2	54	2	54
	Put up the signage and additional environmental changes	Advocate	15 min/months × 12 months × 12 sites	36	975	36	975
Individual & social level	Activities led by an advocate (eg, promote additional signage and idea board content) ^b^	Advocate	75 min × 12months × 12 sites	180	4873	180	4873
Total labor cost				992	24 129	726	19 788
Total non-labor cost ^c^	Sit-stand workstation, footrest, wireless keyboard, & printing (signage starter pack)				23 140		7
Total cost					47 269		19 795
Cost per site/person					3939/134		1650/72

a N=1 unless specified otherwise.

b Participating employees were involved in the activities, but their times/costs spent on the activities during the work hours were not included in the overall intervention cost estimates.

c Quantity for the non-labor cost estimate were 354 for sit-stand workstation and footrest, 12 for wireless keyboard, and one ream of paper and ink for printing the signage start pack.

Non-labor costs including workstations ($300/unit), wireless keyboards ($20/unit), material printing (eg, signage starter pack), and footrests ($4.50/unit) were based on actual amounts spent and were tracked from receipts and payment invoices from the worksites. We did not consider the overhead or space costs given all the SMW intervention activities occurred at workplaces where the participating employees regularly performed their daily work activities. In addition, we annuitized costs of the sit-stand workstations using a 5-year straight-line approach.

### Effectiveness measures

The activPAL3c micro accelerometer (PAL Technologies Limited, Glasgow, UK) was used to objectively measure workplace and overall sitting time and workplace LPA. Work periods were standardized to an 8-hour workday (ie, standardized minutes = observed minutes × 480/observed minutes of wear time). We also collected fasting glucose, insulin, triglycerides, HDL and LDL cholesterol, waist circumference, and resting blood pressure to calculate a summary metabolic risk score (CMR) (30) by summing z-scores for each component of the metabolic syndrome (20). In addition, health-related quality of life was assessed using the Short-Form 12 (SF-12), which consists of two scales; one reflecting mental functioning (the mental health-component scores, MCS-12) and the other physical functioning (physical health-component scores, PCS-12) (31). A SF-12 score ranges from 0–100 with higher scores indicating better health. All assessments were completed at baseline and 3- and 12-month follow-up.

### Health utility

Health utility (EQ-5D) (32) was mapped from the SF-12 score at 12 months using a published algorithm based on a representative US population: EQ-5D = 0.057867 + 0.010367 × PCS-12 + 0.00822 × MCS-12 – 0.000034 × PCS-12 × MCS-12 – 0.01067 (33). Of note, the EQ-5D estimation did not include the number of chronic conditions or family income presented as percent of the poverty line- which were not collected as part of the study protocol. Moreover, because the CEA analysis was conducted using a one-year time horizon, the derived health utility weight at 12 months would be equal to the summation of quality-adjusted life year (QALY), in our case (ie, QALY=health utility weight × 1 year).

### Lost work productivity

The Work Productivity and Activity Impairment (WPAI) Questionnaire (34) was assessed in participants at baseline and 12-month follow up and was used to derive work productivity loss due to a health problem (presenteeism) and absence from paid work due to sickness (absenteeism). Absenteeism was measured by the hours the respondent missed because of illness during the past 2 weeks. Efficiency scores (ie, the degree to which health problems effected productivity while working) were also collected and rated on a scale from 0 (no effect) to 10 (completely prevented me from doing my daily activities). We estimated presenteeism by multiplying the total hours worked in the past two weeks by the efficiency score divided by 10 (35). We then calculated an overall measure of lost work productivity (in hours) in the past two weeks by summing absenteeism and presenteeism across the study arms.

### Statistical analysis

We used linear mixed models to evaluate within-group changes and between-group differences of SF-12 quality of life scores (MCS-12 and PCS-12, respectively), presenteeism, sickness absence, and overall work productivity using the baseline and 12-month outcomes. The group randomized design was accounted for using a random effect for site nested within treatment (MOVE+ versus STAND+). Models were adjusted for baseline values of the respective outcome and a priori selected covariates: age, sex, race/ethnicity, and baseline body mass index (BMI), as performed for the primary outcomes of the trial (21).

### Cost-effectiveness analysis

We conducted the CEA using an incremental cost-effectiveness ratio (ICER) which was calculated as the incremental costs per additional unit increase in effectiveness outcomes between MOVE+ and STAND+ regardless of the significance of the between-group differences. This was based on the justification that adoption decisions should be based on the mean net benefits irrespective of whether differences are statistically significant (arbitrary rules of inference) (36). We calculated the ICER in terms of effectiveness outcomes of reduction of workplace and overall sitting time, workplace LPA, CMR score, and QALY gain derived from mapped EQ-5D, assuming that baseline utility weights were comparable between the intervention and control groups.

### Return on investment analysis

We calculated the return on investment analysis according to the employer’s perspective as: benefit-cost ratio (BCR)=benefits/costs. If BCR is >1, the financial return of the investment was positive. Specifically, costs were defined as SMW intervention delivery costs, whereas benefits were defined as the difference in lost productivity costs (ie, sickness absence from paid work and presenteeism due to health problem) between MOVE+ and STAND+ at the 12-month follow-up, with positive benefits indicating reduced spending/productivity gain. The lost productivity costs were defined as the costs associated with time lost from paid work (ie, absenteeism and presenteeism) due to a health problem, and the human capital approach using a national average hourly wage was applied for the estimation. We extrapolated the adjusted difference in the lost work productivity in the past two weeks (results of linear mixed models) between STAND+ and MOVE+, over a 52-week (ie, one year) time period. To derive the overall cost of lost work productivity over a one-year time period, we then multiplied the overall lost work productivity (measured in hours) with a value of $27.07 per hour (27).

### Sensitivity analysis

**Figure 1:**
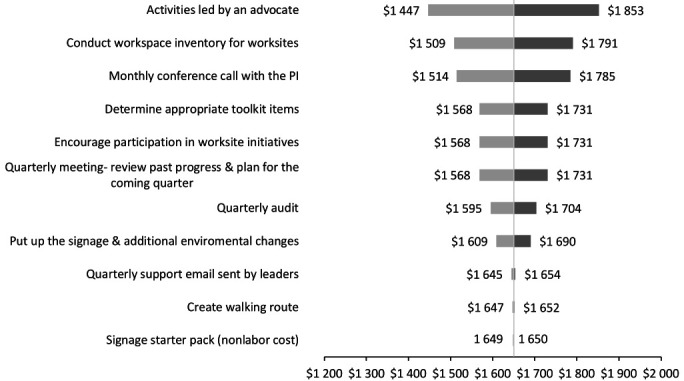
One-way sensitivity analysis for the total costs per site of the MOVE+ intervention by intervention activities. Each row shows the changes in costs, across the range of the hourly wage rates, from the original total costs ($1650). [PI=principal investigator.]

The MOVE+ intervention consisted of only fixed costs (table 1), which did not vary by the number of participants. In contrast, the STAND+ intervention included fixed and variable (ie, workstation and training to use the workstation) costs, the latter fluctuating by the number of participants. Consequently, we conducted a one-way deterministic sensitivity analysis of the total cost of the MOVE+ intervention to estimate the replication costs if the program was implemented under different conditions. We varied the unit costs (ie, hourly wage rates) of the intervention activities outlined in table 1 by ±50% to derive the lower and upper bounds of the plausible values for the activities (37). We presented the sensitivity analysis results in terms of the total intervention delivery cost per worksite with additional information on variable costs per person to facilitate the decision making for employers who may want to continue or adopt the STAND+ or MOVE+ interventions at their workplace, using a tornado diagram (figure 1).

Analyses were performed according to the intention-to-treat principle using Stata 16.0 (StataCorp LP, College Station, TX) or Microsoft Excel.

## Results

### SMW intervention delivery costs

Table 1 presents the breakdown of time and costs for the STAND+ and MOVE+ interventions. The overall intervention costs totalled $47 269 and $19 795, respectively, which translated to $134 and $72 per participating employee, and $3939 and $1650 per site, for STAND+ (N=12 worksites; N=354 participants) and MOVE+ (N=12 worksites; N=276 participants), respectively. Without considering workstation associated activities, organizational-level strategies accounted for 33% of the labor costs, related to individual- and social-level (25%), environmental-level (22%), and policy-level (20%) strategies. The average hours that an advocate at a worksite spent on these strategies were estimated at 40 hours over the 12-month study period. In addition, the time that participating employees spent engaging with intervention activities during the work hours were estimated at 15.75 hours (workstation installation, coaching session, and activities led by an advocate) per person for STAND+ and 15 hours (activities led by an advocate only) for MOVE+ during the 12-month study period. The total non-labor costs were estimated at $23 204 (49% of the overall intervention delivery costs) for STAND+, including 354 workstations and footrests, 20 wireless keyboards, and a signage starter pack, whereas it was $7 for MOVE+ (table 1).

### Effectiveness

STAND+ significantly reduced workplace sitting time compared to MOVE+ [59.2 minutes/8-hour workday; 95% confidence interval (CI) 43.8–74.6 minutes] as well as total sitting time (47.7 minutes, 95% CI 31.7–63.6) at 12-months. However, there were no statistically significant differences in the outcomes of workplace LPA and CMR scores between study conditions regardless of the positive direction observed [the full account of effectiveness outcomes have been published elsewhere (21)]. STAND+ was also associated with non-significant changes in work productivity loss at 3 (4.58 hours, 95% CI -1.06–10.21) and 12 months (-0.03 hours, 95% CI -4.16–4.09) as well as increases in PCS-12 (0.61, 95% CI -1.68–1.89) and MCS-12 scores (0.54, 95% CI -1.30– 2.37) at 12 months compared to MOVE+. Changes in work productivity loss were modestly attenuated at 12 months. Similar results were found for the analysis targeting the dysglycemic participants (N=122), STAND+ had a greater non-significant work productivity loss (1.25 hours, 95% CI -5.57–8.07) and a significantly lower quality of life-mental health score (MCS-12= -3.19, 95% CI -6.05– -0.34; P=0.028) at 12-month follow-up, compared to MOVE+. The adjusted and unadjusted differences of the outcome variables between STAND+ and MOVE+ at 12 months are provided in table 2.

**Table 2:** Adjusted and unadjusted group differences of outcome variables between STAND+ and MOVE+ at 12 months. [CI=confidence interval; CMR=summary continuous metabolic risk; BMI= body mass index.]

Outcome	Adjusted^a^		Unadjusted^b^	
	
	Difference	95%CI	Difference	95%CI
Changes in workplace sitting time (minutes)	-59.2	-74.6- -43.8	-59.7	-77.5- -41.8
Changes in workplace light-intensity physical activity (minutes)	2.2	-0.9- 5.4	2.2	-0.7- 5.0
Changes in total sitting time (minutes)	-47.7	-63.6- -31.7	-47.8	-65.3- -30.4
Changes in CMR score	-0.03	-0.10- 0.04	-0.03	-0.11- 0.04
Changes in productivity loss (hours)	-0.03	-4.16, 4.09	0.24	-3.71- 4.19
Changes in sickness absence (hours)	0.45	-1.63, 2.53	0.76	-1.28- 2.80
Changes in presenteeism (hours)	-0.47	-3.82, 2.88	-0.52	-3.70- 2.67

a Group differences derived from the linear mixed models adjusted for baseline values of the respective outcome and age, sex, race/ethnicity, and baseline BMI.

b Group differences derived from the linear mixed models without adjustment.

### Cost-effectiveness and return on investment analysis

The CEA results in terms of effectiveness outcomes regardless of significance are reported in table 3. The ICER was $1.0 and $1.3 for additional minute reduction in workplace sitting time and total sitting time, $28 for additional unit increase of workplace LPA, and $2060 for additional CMR score reduction. Moreover, the ICER, comparing STAND+ with MOVE+, was $4656 per additional QALY gained. When including the work loss associated costs from participating employees, the ICER was $1.4 (95%CI $1.1–$1.9) and $1.7 (95% CI $1.3–2.6) for additional minute reduction in workplace sitting time and total sitting time, $37 (95% CI $15–91) for an additional unit increase of workplace LPA, and $2737 (95% CI $821–2053) for an additional CMR score reduction, and $6186 for additional QALY gained. The BCR was calculated as $0.34 for every $1 invested. In other words, for every dollar spent $0.34 is returned.

**Table 3:** Cost-effectiveness results of STAND+ relative to MOVE+. [CMR=summary continuous metabolic risk; ICER=incremental costeffectiveness ratio; LPA=light-intensity physical activity].

Study outcomes	Incremental cost (∆C) $	Incremental effectiveness (∆E), mean (95% CI) ^a^	ICER $ (∆C/∆E), mean (95% CI) ^b^
Reduction in workplace sitting time	62	59.2 (43.8–74.6)	1.0 (0.8–1.4)
Changes in workplace LPA	62	2.2 (-0.9–5.4)	28
Reduction in total sitting time	62	47.7 (31.7–63.6)	1.3 (1.0–2.0)
Reduced CMR score	62	0.03 (-0.04–0.10)	2060
Changes in mapped EQ-5D score	62	0.013 (-0.002–0.028)	4656

a To indicate the reduction of workplace sitting time, total sitting time, and CMR scores as positive benefits of intervention and reflected in the ICER calculation, we presented the effect sizes as a positive observation by including the term of “reduced” in front of study outcomes.

b We did not present 95% CI for ICER for study outcomes with a non-significant effect size due to a potential negative ICER.

### Sensitivity analysis

The one-way sensitivity analysis results are presented in figure 1 with a tornado diagram that summarizes the effect of variation on intervention activities one at a time on the total intervention cost estimates per site for the multilevel behavioral intervention without a sit-stand workstation (MOVE+). The intervention strategy with the greatest impact on the total costs per site was *activities led by an advocate*, as the estimates range from $1447–1853 per site, following *conduct workspace inventory for worksites*. In addition to cost variations resulting from labor activities, the other potential cost uncertainty was the cost of the sit-stand workstation and footrest, estimated at $61 per participant per year (for STAND+ only), with a 5-year depreciation, and the associated installation and training (~45 minutes), estimated at $12 per person.

## Discussion

To the best of our knowledge, this is the first study to examine the cost-effectiveness of the addition of sit-stand workstations relative to a multi-level intervention to reduce workplace SB. Other studies that have assessed the cost-effectiveness of multi-level interventions have either had no intervention (14) or a usual care group (eg, less intensive intervention) as the comparator (18). By comparing two active intervention programs (STAND+ versus MOVE+) – one with and one without a sit-stand workstation – our CEA results indicated ~$1 for an additional minute reduction in workplace sitting time. In comparison, Gao et al (14) compared a similar workplace SB intervention following the ecological framework to no intervention and indicated an ICER of AU$9.2 for an additional minute reduction in workplace sitting time at 12-months [equal to $6.3, converted to purchasing power parity dollars for 2020 using country specific inflation rates (38)]. Moreover, it was £16.8 ($24.5) for the SMArT Work intervention conducted in UK (17). In contrast, in the Netherland study, Ben et al (18) indicated that the intervention was less costly but also less effective compared with the control.

The cost and cost-effectiveness analysis of SMW was completed to inform the adoption decision-making process for workplaces interested in reducing SB. Our cost analysis results showed an employer would need to invest a fixed baseline cost of $1650 for the one-year intervention regardless of how many employees participated, and a variable cost of $73 per person ($61 for workstation and footrest and $12 for workstation installation and training). The variable costs of a sit-stand workstation may be further reduced through bulk orders, shared-use, and so forth (14). Furthermore, in contrast to previous works (14, 17, 18), we compared the cost and cost-effectiveness of a multi-level behavior intervention with a sit-stand workstation (STAND+) with a multi-level intervention without a sit-stand workstation (MOVE+). The sensitivity analysis of fixed costs provided sufficient information on the total intervention delivery cost per worksite with additional information on variable costs per person, to allow employers to make an informed decision of whether to continue or adopt the individual STAND+ or MOVE+ intervention.

Although workplace SB interventions were mostly multi-component interventions, they varied in degrees of intensity, which may impact the observed effect size. In the current study, the magnitude of the effect of the reduction of workplace sitting time was estimated at 59.2 minutes per workday, which is comparable to the 46.8 and 41.3 minutes in Gao et al (14) and Munir et al (17), respectively, with similar intervention components. However, the effect was smaller in the study conducted by Ben et al (18) (-0.11h/16h day (=~6.6 minutes) (39) at 8 months), potentially attributed to the fact that the intervention components were less intensive (partial replacement for workstations for some employees and group coaching session). Moreover, the cost composition of work productivity loss also varied by studies. Similar to the present study, both Munir et al (17) and Ben et al (18) included both presenteeism and absenteeism as a sum of the lost productivity cost; whereas Gao et al (14) only included absenteeism costs. This operationalization may underestimate the costs as a result of work productivity loss or underestimate the potential work productivity gain from the multilevel intervention. In addition, the length of the intervention period (ie, the primary endpoint) was 12-month in Gao et al (14), Munir et al (17), and the present study; whereas it was 8-month in Ben et al (18).

For the ability to compare across studies, we described the intervention cost as a unit cost (per participant) as the total intervention costs differed in study scope and size, resulting in great variability (40). Intervention costs were estimated at $297, $366, and $870 per participant, in Gao et al (Australia) (14), Ben et al (The Netherlands) (18), and Munir et al (the UK) (17), respectively, relative to $134 per participant in STAND+. The cost of the sit-stand workstation was the major cost driver of the multi-level interventions (18), and their types and costs varied across studies. Moreover, in the present study, we annuitized the cost of the sit-stand workstation over 5 years which is consistent with past research (14). As such, the overall intervention cost was reduced from $375 to $134 per person. However, this procedure was not conducted in Ben et al (18) and Munir et al (17). Nevertheless, our results remain comparable even without depreciation. Finally, we did not include costs of activity trackers (ie, activPAL3c) and/or self-monitoring and prompt tools (17, 18) nor participant time (ie, opportunity cost/work loss) (14, 17) in the cost estimate of the intervention costs as done in other studies. We considered the cost of activPAL3c as research-related and took the employer perspective for the CEA analysis, where participant time was not included. Of note, the impact of participant time (ie, opportunity costs) was considered minimal (45 minutes per employee in the STAND+ arm only for the workstation installation and training), or cancelled each other out between STAND+ and MOVE+ (approximately 15 hours extra engagement times with activities led by advocates at worksites over the 12-month intervention period for both study arms) if taking a limited societal perspective.

In contrast to the ICER findings and different from the positive return on investment (amount of money return per $1 invested) reported in Munir et al ($5.2) (17) and Ben et al ($4.2) (18) as a result of an increase in work productivity at 12-months, our results indicated a negative return on investment of $0.34. There was large variability in the cost estimate of presenteeism and absenteeism across studies evaluating workplace SB interventions with studies indicating a null/negative association between workplace SB intervention and presenteeism/absenteeism outcomes (7). This observation may be attributed to the fact that the presenteeism/absenteeism outcomes were measured using self-report [it was collected through employer recorded data in Munir et al (17) and Ben et al (18)] with employees potentially not wanting to report lower performance during work due to health problems; or cost estimate approach for the cost of lost work productivity used in the studies [the friction cost approach in Ben et al (18); and the human capital approach using the individual employee wage-banding information in Munir et al (17)]. Nevertheless, these findings are of importance given that presenteeism and absenteeism may be of great interest to employers when considering whether or not to adopt the workplace SB interventions (7). Future research should examine work productivity-related outcomes with reliable instruments or data sources [eg, company administrative record (18)], and consider the trade-off between the frequency of measurement, recall period, and the potential recall bias and inaccuracy, given that no standard exists on how long the recall period should be (35, 41–44). Another explanation of negative return on investment in the present study may be that our study population was relatively healthy- the average BMI was 29.3 kg/m^2^ and only ~19% of participants reported having a previous diabetes diagnosis or a fasting blood glucose ≥100 mg/dL (21). It might be that healthy employees’ productivity level is higher than employees’ with special health conditions and thus may experience smaller marginal impact from health improvement.

In addition, our results indicated that the ICER was $2737 for an additional CMR score reduction. A unit change in the CMR score was one standard deviation (SD). Other similar metrics of cardiometabolic health, in large population-based cohorts, have demonstrated that a 1 SD reduction was associated with 1.8 lower odds of incidence of cardiovascular disease and 5.1 lower odds of incidence of type 2 diabetes (45). The reduction of CMR scores may potentially in turn decrease associated healthcare utilizations and costs. Finally, the ICER was $4656 for an additional QALY gained with a one-year time horizon, which is considered cost-effective with the willingness-to-pay threshold value of $50 000 per QALY (46, 47).

Despite the fact that QALY is recognized as a standard metric of health outcome in CEA, the data may not be widely available from the randomized trials of the interventions. Moreover, worksite decision-makers may see it challenging to conceptualize QALY and the associated interpretation. Accordingly, in addition to ICER, presenting other generic cost-effectiveness outcomes (eg, costs per workplace sitting time reduced between STAND+ and MOVE+) simultaneously, may improve the uptake of the program adoption or maintenance by increasing decision makers’ comprehension of the CEA results.

### Limitations

First, the present study may suffer from issues related to recall bias, given all the cost data were collected retrospectively by interviewing the advocates, project coordinator and staff. Moreover, because participants were generally in good health and able to safely reduce sitting and increase LPA (one of the eligibility criteria), we may underestimate the effect of absenteeism and presenteeism. Participants may not have been able to recall the events of sickness absence or have a higher self-rated efficiency score at work. Second, given that the SMW trial was a cluster randomized controlled trial, where the worksite was the unit of randomization, employees within a worksite were free to choose whether to participate in the intervention program. It is likely that the addition of a sit-stand workstation may influence employees’ willingness to participate in the program (ie, selection bias). Third, although we derived the presenteeism and absenteeism data using the validated questionnaires, there is still bias associated the self-reported nature, and the retrospective survey questions assessing for the preceding period of past 2 weeks, completed three times across 12-month period. Fourth, we used the past 2-week productivity change between baseline and 12-month follow up to estimate the productivity gain between the intervention groups across entire 12-month period, as opposed to derived from employer data (ie, company records) (17, 18) due to data unavailability. Moreover, we recognize the limitation that a short-term timeframe (ie, one year) was applied to the current economic evaluation due to study protocol of the main trial. Studies have suggested that a longer time horizon (eg, model-based economic evaluations), is warranted (19). Fifth, we recognize that it is a norm to present results of a sensitivity analysis related to the ICER (comparing STAND+ to MOVE+) in the typical CEA. However, because (i) the costs estimated from the current study were obtained from an aggregated form (we did not have the individual cost data for either the worksite or the participants); (ii) the only cost difference between STAND+ and MOVE+ was the sit-stand workstation and footrest; and (iii) the willingness-to-pay threshold for the SB interventions has not been established yet (19), our capability was limited in conducting sensitivity analysis of the ICER. Finally, the trial used the generic health status measure (ie, SF-12), however, it cannot be used in CEA due to the lack of preference (ie, health utility weight) for health state. Alternatively, we used a validated mapping algorithm to convert SF-12 to preference-based EQ-5D index scores and applied derived score in the CEA. Nevertheless, the imputation of health utilities may potentially suffer from biased estimates relative to the health utility derived directly from the use of the preference-base health-related quality of life instruments (e.g., EQ-5D).

### Concluding remarks

In the study, we presented a detailed costing process of the multi-level intervention on SB to facilitate future comparative cost analysis. Furthermore, the multi-level intervention with sit-stand workstations holds the potential to be widely implemented within worksites and was effective in reducing workplace sitting time. Future research examining work productivity outcomes in terms of cost-benefits for employers is warranted.
